# Genic microsatellite marker characterization and development in little millet (*Panicum sumatrense*) using transcriptome sequencing

**DOI:** 10.1038/s41598-021-00100-4

**Published:** 2021-10-18

**Authors:** Hiral Desai, Rasmieh Hamid, Zahra Ghorbanzadeh, Nishant Bhut, Shital M. Padhiyar, Jasminkumar Kheni, Rukam S. Tomar

**Affiliations:** 1 Department of Biotechnology, Junagadh Agricultural University, Junagadh, Gujarat India; 2 Department of Plant Breeding, Cotton Research Institutes of Iran (CRII), Agricultural Research, Education and Extension Organization (AREEO), Gorgān, Iran; 3 Department of Systems Biology, Agricultural Biotechnology Research Institute of Iran, (ABRII), Agricultural Research Education and Extension Organization (AREEO), Karaj, Iran; 4 Main Oilseeds Research Station, Junagadh Agricultural University, Junagadh, Gujarat India

**Keywords:** Biotechnology, Genetics, Plant sciences, Systems biology

## Abstract

Little millet is a climate-resilient and high-nutrient value plant. The lack of molecular markers severely limits the adoption of modern genomic approaches in millet breeding studies. Here the transcriptome of three samples were sequenced. A total of 4443 genic-SSR motifs were identified in 30,220 unigene sequences. SSRs were found at a rate of 12.25 percent, with an average of one SSR locus per 10 kb. Among different repeat motifs, tri-nucleotide repeat (66.67) was the most abundant one, followed by di- (27.39P), and tetra- (3.83P) repeats. CDS contained fewer motifs with the majority of tri-nucleotides, while 3′ and 5′ UTR carry more motifs but have shorter repeats. Functional annotation of unigenes containing microsatellites, revealed that most of them were linked to metabolism, gene expression regulation, and response to environmental stresses. Fifty primers were randomly chosen and validated in five little millet and 20 minor millet genotypes; 48% showed polymorphism, with a high transferability (70%) rate. Identified microsatellites can be a noteworthy resource for future research into QTL-based breeding, genetic resource conservation, MAS selection, and evolutionary genetics.

## Introduction

Minor millets belong to the Poaceae family, and are small-grained cereal crops known for their climate-resilient cultivation^1^. Small millets or “Minor Millets” are raised in the semi-arid tropical realms of Africa and Asia^2^. They include foxtail millet (*Setaria italica*), proso millet (*Panicum miliaceum*), barnyard millet (*Echinocloa frumentacea*), little millet (*Panicum sumatrense*), kodo millet (*Paspalum scrobiculatum*), and finger millet (*Eleucine coracana*). They are one of the most important crops with many nutritional benefits. They have a good amino acid profile, are rich in micronutrients e.g., calcium, zinc, iron, and iodine, and are gluten-free. They have excellent health benefits for patients who have atherosclerosis, diabetes, heart attack, blood pressure, migraine, and asthma. Its high fiber content prevents gallstone formation^3^. Minor millets are usually neglected in the society. Thus, very little information is available about their genome, transcriptome, and other essential aspects, which is necessary for initiating new research programs for food security globally^4^. Few genetic resources developed in finger millet, foxtail millet, proso millet, and Japanese barnyard millet^5^. Progress towards the desired goals necessitates modern breeding approaches based on DNA-based molecular markers^6^, particularly co-dominant marker systems^7^. Recent, crop improvement programs widely done using molecular breeding or marker-assisted breeding approach that utilizes molecular marker like EST-SSR or Genic SSR and genome-wide SSR marker with the use of publicly available genomic and transcriptomic resources available^8^.

Among the molecular markers, SSRs are the most commonly used molecular markers; they are also known as microsatellites and are widely distributed throughout the genome, and are extremely polymorphic, chromosome-specific, and frequently inherited in a Mendelian co-dominant fashion^9^. In contrast to genomic SSR markers, Genic SSRs or Expressed sequence tags microsatellites (eSSRs) are acquired through expression sequence tags generated by converting gene transcripts into cDNA^10^. As they originate from coding regions, eSSRs markers are preferred for studying plant species^11^. Genic SSRs are found within gene sequences and are conserved; hence they can be applied to define alleles associated with important agronomical traits^12,13^.

**Figure 1:**
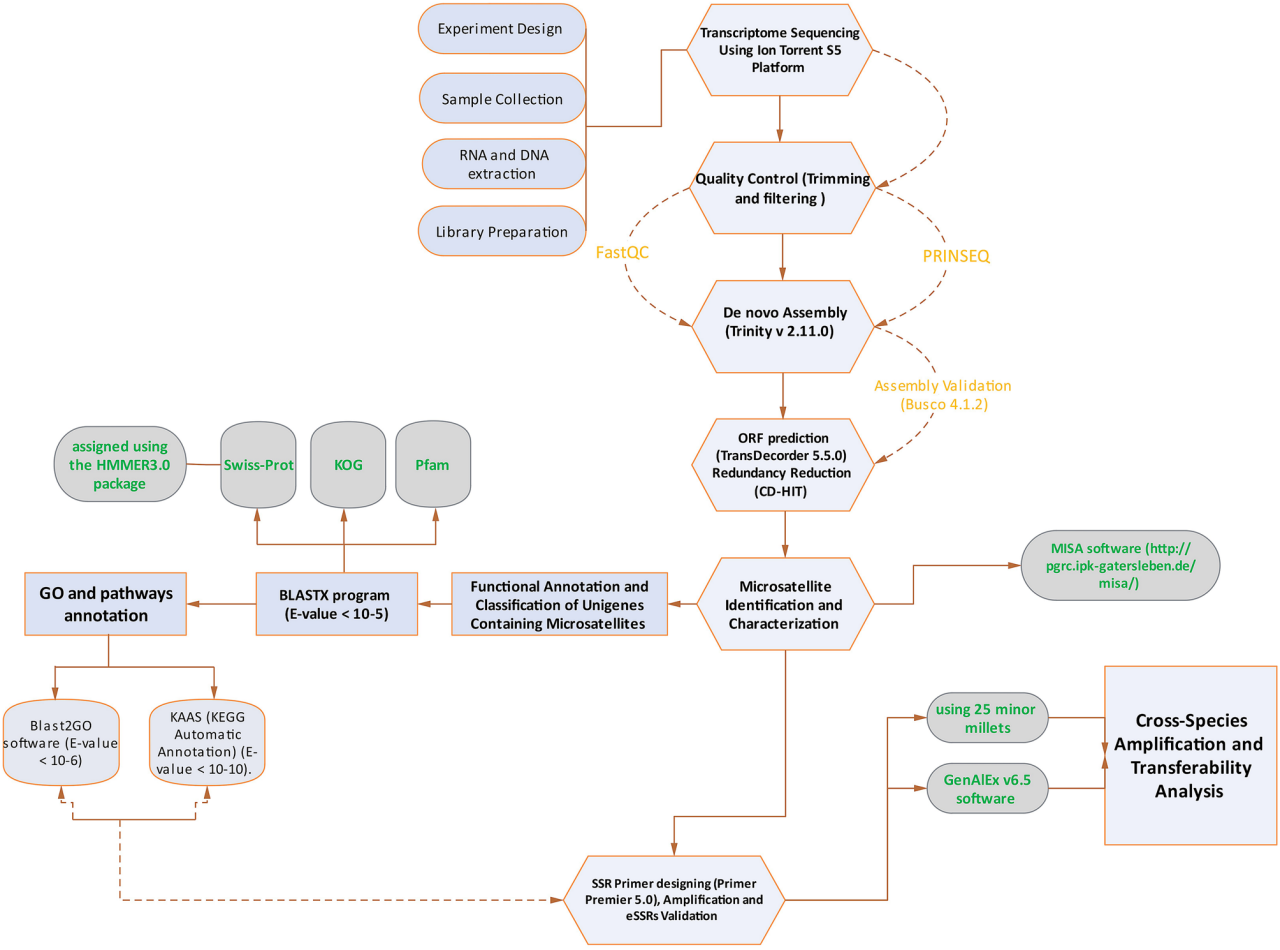
A detailed workflow performed in identification and validiation of gSSR in Little millet (*Panicum sumatranse*), this figure was created using ggplot2 R-package.

Furthermore, because of their high polymorphism, multi-allelic nature, reproducibility, and transferability, eSSRs are widely used as a robust molecular marker in marker-assisted breeding for crop improvement^14,15^. Traditionally, SSR development is labor-intensive, like cloning DNA and building a library, and produces significantly fewer SSRs than next-generation sequencing (NGS) technology^16^. The added value of NGS technologies, particularly transcriptome sequencing, is that they can provide a plethora of high-quality and cost-effective sequences in a short period^17^. RNA sequencing (RNA-seq) helps us achieve details on functional genes that can be applied to detect eSSR markers in a reliable and high-throughput manner^10,18^. Applying RNA-seq techniques, eSSRs have been identified in several plant species, including Mulberry^19^, bean^20^, avocado^18^, turmeric^21^, apple^22^, barley^15^, cotton^23^, wheat^24^, Coriander^25^, peanut^13^, and Pistacia^26^. In this research, we used transcriptome sequencing to mine microsatellite loci from expressing sequences of little millet. The goals of this research are (1) to identify the frequency, distribution, and the role of genic microsatellites in the transcriptome of little millet; (2) establish polymorphic microsatellite markers and validate their polymorphism degree; and (3) assess cross transferability between/among other millet spices. The current study's flowchart is showed in Fig. 1.

## Materials and methods

### Plant materials

Little millet seed material was collected from the Indian Institute of Millets Research, Rajendranagar (Hyderabad, Telangana, India) and planted in the Department of Biotechnology, Junagadh Agricultural University's controlled climatic conditions of 70% relative humidity and 20/15 °C (14 h/10 h) day/night temperature regime. Leaves from the vegetative, flowering, and maturity stages were collected for RNA sequencing. All specimens were frozen in liquid nitrogen, immediately, and kept at − 80 °C until used. Young leaves from 25 genotypes (Table S1) were also collected for DNA extraction, which then used in the EST-SSR marker analysis. The collection of seeds and the complete experiment was carried out according to the national guidelines^27^.

### RNA extraction and cDNA library preparation

The RNeasy® Plant mini kit (Qiagen, Valencia, CA) was utilized to isolate total RNA from leaf samples obtained at the vegetative, flowering, and maturity stages, according to the manufacturer's instructions. The consistency and concentration of RNA were determined using a 1.2 percent agarose gel electrophoresis and a Qubit® 2.0 Fluorometer (Thermo Scientific, USA). The RNA Nano 6000 Assay Kit was also used to determine the integrity of the RNA. mRNA isolation from total RNA was carried out using oligo-dT-attached magnetic beads (Dynabeads® mRNA DIRECT™ Micro Kit), and Ion total RNA seq-kit v2 was used to create cDNA libraries (Thermo Fisher Scientific). For cDNA library preparation, purified mRNA randomly fragmented into short fragments (~ 200 bp) by RNaseIII enzyme then subjected to Ion adapter v2 hybridization, ligation and cDNA was synthesized and amplified as per manufacturer’s guidelines. After amplification, cDNA libraries were selected for target fragments of ~ 200 bp on 2% Agarose EGelTM (Thermo Fisher Scientific) and stored at − 20 °C until used^10,28^. The selected ~ 200 bp cDNA library was subjected to emulsion PCR (Ion OneTouch™ 2 System, Thermofisher Scientific, USA), followed by enrichment of template positive bead recovery. Sequencing was conducted in an Ion S5 Machine (S5TM, Life Technologies). During the library preparation, each sample was assigned a unique molecular barcode.

### Reads processing and assembly

PRINSEQ (0.20.4) was utilized for omitting low-quality sequences after FastQC (v0.11.9); the quality of the reads (if the reads had more than 10% nucleotide bases with Q value 25) were checked. Trinity (v2.13.2) reconstructed and assembled the high-quality reads into unigenes, using the default parameter^25^. To benchmark completeness of individual and combined *P. sumatrense* transcriptome assembly, the Benchmarking Universal Single-Copy Orthologs (BUSCO) version v5.2.2 was used with the default E-value cut-off of 1e−03 against the ortholog set of Embryophyta_odb9 lineage (creation date: 2020-09-10, number of species: 70, and number of BUSCOs: 255) from OrthoDB v9^29^. Further, TransDecoder (v. 5.5.0) was used for estimating the coding sequence present in Master assembly with the default parameters^30^. The single best open reading frame (ORF) per transcript, longer than 200 peptides were selected. Further CD-HIT-EST program (v. 4.8.1) was used to reduce redundancy in transcript sequence and generate Unigene^31^. Then standalone blast with a command line for Basic Local Alignment Search Tool (BLAST) against the Uniprot database for functional annotation was used^32^.

### SSR marker identification and primer design

The MIcroSAtellite (MISA) Identification Tool Perl script (http://pgrc.ipk-gatersleben.de/misa/) was used to find simple sequence repeat regions. The assembled transcriptome was screened for di-, tri-, tetra, Penta-, and hexanucleotide repeat motifs. Primer 3.0 (http://fokker.wi.mit.edu) was used to design primers from sequences containing SSRs. In addition, the following parameters were considered for primer designing: primer length 18–28 bp optimum length 20 bp; Tm-55–63 °C, with an optimum of 60 °C; GC content 40–60%, optimum value 50%; maximum Tm difference between forward and reverse primer 1.5 °C and product size range 100–300 bp, 150 bp being the ideal size^23,33^.

### Assigning functions to unigenes containing microsatellites

Functional annotation of SSR containing sequence carried out using standalone blast with NCBI non-redundant Nr database (e-value cut-off 10^−6^), Swiss-Prot (A manually annotated and reviewed protein sequence database), KOG (Clusters of eukaryotic Orthologous Groups), and Pfam (Protein family, assigned using the HMMER3.0 package)^30^. Furthermore, the Blast2GO software with a cut-off E value of 1e^−5^ was used to assign GO (Gene Ontology) annotation on all unigene containing microsatellite motifs. Also, KEGG (KAAS, http://www.genome.jp/kegg) was used to perform metabolic pathways analyses (E-value 10^−10^).

### DNA extraction, PCR, and eSSR validation

A total of **50** randomly selected sets of primers were selected and synthesized (Merck, India). Genomic DNA isolation was illustrated using DNeasy plant Mini Kit (Qiagen, Valencia, CA) from young leaves samples of five little millet, five proso millet, five Kodo millet, five barnyard millet, and five finger millet genotypes (Table S1). The polymerase chain reaction was carried out using the following PCR components: contained 1μL template DNA (50 ng), 1.0 μL forward primers, 1.0 μL reverse primers 2 μL 10 × Taq buffer + MgCl2 (15 mM), 2 μL dNTP (2 mM), 0.4 μL Taq polymerase (Promega 5U μL^−1^) and 2.6 μL sterile distilled water. PCR reaction was performed in Veriti thermal cycler (Applied Biosystems, USA) with the following condition: Initial denaturation 94 °C for 3 min, denaturation 94 °C for 30 s, annealing 30 cycles 58–59 °C for 30 s and final extension at 72 °C for 7 min. Each primer's PCR product was visualized at 5 V/cm using 3 percent MetaPhor Agarose Gel Electrophoresis (MAGE) (Biowittaker, USA) in TBE buffer^34,35^. The gel was stained with EtBr (0.5 mg/ml) after the run^36^, and Agarose gel scanning was carried out in a Gel Documentation system (Syngene, India). A 50 bp Plus DNA Ruler (Thermofisher, USA) was used as a molecular size standard.

### Polymorphism estimation and cluster analysis

The **24** polymorphic eSSR products were scored manually as ‘1’ (presence) and ‘0’ (absence). The PIC was calculated using the score from the polymorphic loci and the formula PIC = ∑1 − gpi (where pi is the frequency of ith allele for each locus)^37^. The Popgene software version 1.31 was used to compute the observed heterozygosity (Ho) and expected heterozygosity (He). A matrix for genetic similarity was generated using the NTSYSpc 2.1 software^38^. UPGMA algorithm in NTSYS pc software was used to depict similarity coefficients and genetic similarity among millet genotypes. Dendrogram and All the other graphs were prepared using R software version R-4.1.1^39^ by ggplot277 R-packages^40,41^.

## Results and discussion

### De novo assembly and characterization of unigene

**Figure 2:**
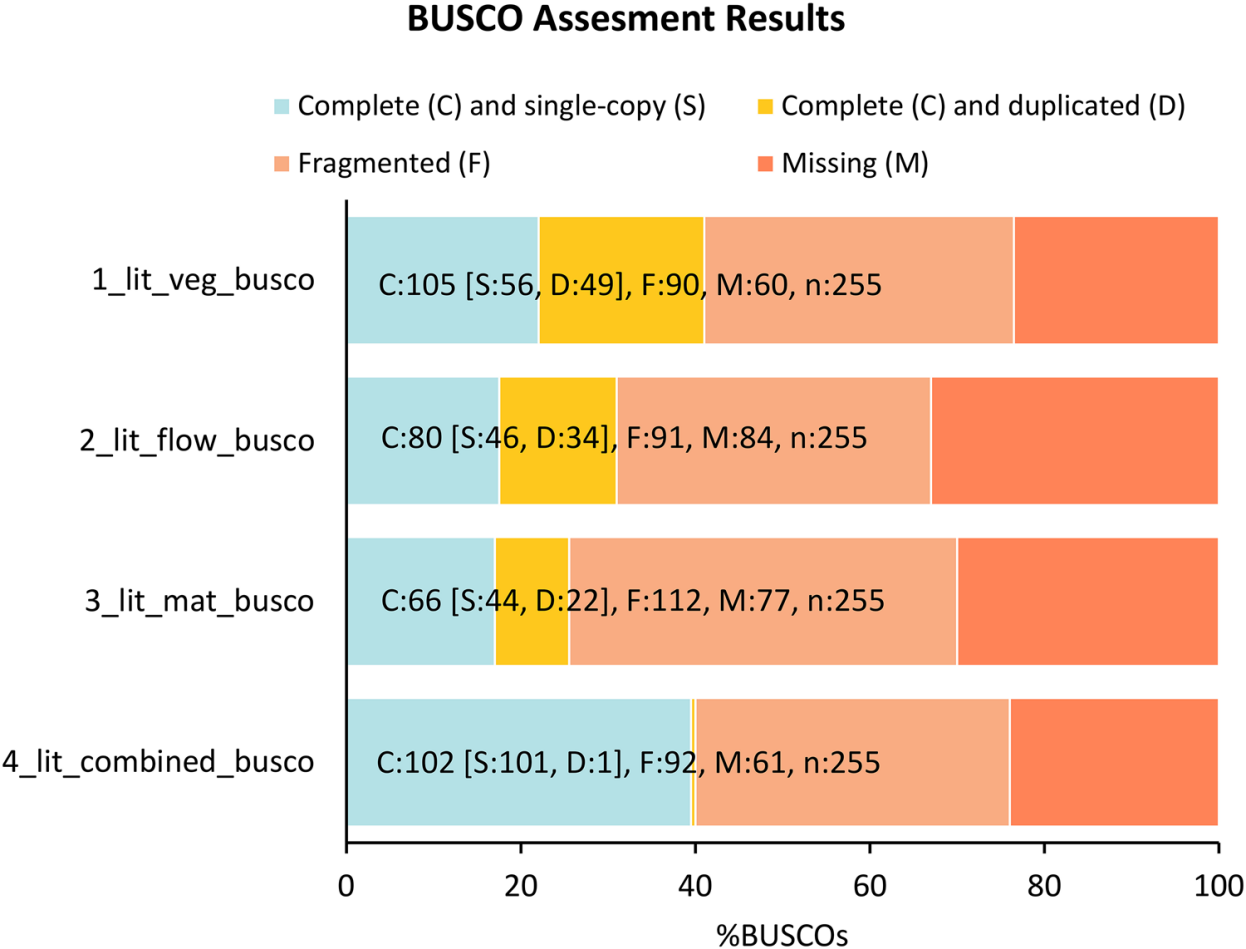
Transcriptome completeness as determined by Benchmarking Universal Single-Copy Orthologous (BUSCO) version 4.1.1 against the ortholog set of Embryophyta_odb9 lineage (creation date: 2020-09-10) from OrthoDB v9.

In this study 11.4, 7.6, and 10.5 million reads with average read length 158 bp, 165 bp, and 150 bp were generated in leaf transcriptome at the vegetative stage, flowering, and maturity stage, respectively. Reads with Q25 bases (i.e., reads with a base quality ≥ 25) were selected as high-quality reads, through additional analysis (Table 1). Finally, using Trinity, a total of 29,629,186 (81.36 P) reads were assembled into 47,358 unigenes. All unigenes were classified based on the length of the sequences, and the average length of the unigenes was estimated to be 521.72 bp. The majority of the reads (91.7%) were in the 200–1000 bp range. There were 25,743 unigenes less than 1000 bp in length and 5,038 with more than 1000 bp (16.36P) (Fig. S1). The completeness of transcripts was determined by comparing them to universal single-copy orthologs (BUSCO). The percentage of detected BUSCOs is represented on the x-axis (Fig. 2). The powder blue diamond illustrates the complete (C) and single-copy (S) genes; the sunglow stands in for complete and duplicated (D) genes; the tacao diamond represents fragmented (F) genes; the coral diamond act for the missing (M) genes. In the vegetative, flowering, and maturity stages, the total number of core genes queried were 45,852, 47,851, and 48,370, respectively. BUSCO evaluated the completeness of transcripts. BUSCO assessment of the quality of transcriptome assemblies revealed that individual transcriptome assemblies have low completeness (17.3 to 22%), higher fragmentation (35.3% to 43.9%) and higher missing sequences (23.5 to 33%) compared with the combined assembly. The combined assembly showed high percentage of gene representation (40%), high completeness (39.6%), low percentage of fragmented (36%) and low missing sequences (23.9%). The single-copy BUSCOs accounted for 22 percent, 18 percent, and 17.3 percent of the vegetative, flowering, and maturity stages, respectively. However, the single-copy BUSCOs in the combined assemble was 39.6% with only 0.4% duplicates. Of the total 255, BUSCO groups searched, only 35.3%, 35.7%, 43.9% fragmented BUSCOs, and 23.5%, 33.0%, and 30.2% missing BUSCOs were found in our database, respectively in the vegetative stage, flowering stage, and maturity stage (Fig. 2). All of these findings indicated that our database was complete and ready for further investigation.

**Table 1:** Statistics of RNA sequencing data generated with Ion Torrent S5.

Samples	Total no. of reads	Total nucleotides (bp)	GC (%)	Q25 (%)
Vegetative	11,458,128	1,198,271,216	45.43	80.17
Flower	7,622,224	1,274,719,652	45.25	80.47
Maturity	10,548,834	1,871,180,533	45.45	83.44
Total	29,629,186	4,344,171,401	45.44	81.36

### EST-SSR discovery, frequencies, and distribution

**Figure 3:**
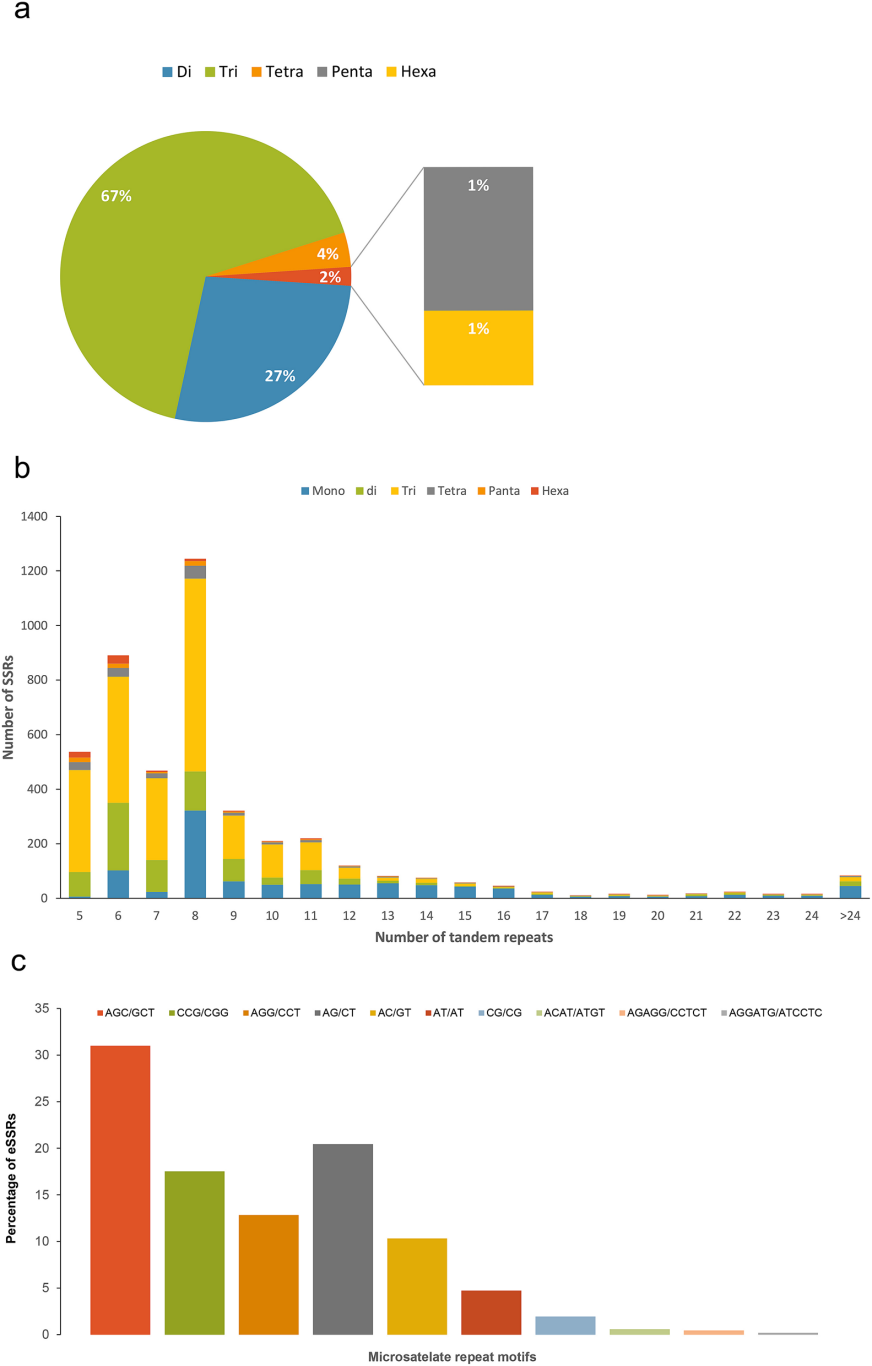
The frequency distribution of the SSRs identified in transcriptome dataset. (**a**) Distribution of the total number of EST-SSRs in different classes of repeat type. Di-, tri-, tetra-, penta- and hexa-nucleotide repeats were analyzed. (**b**) Distribution of microsatellite motif types and tandem repeat numbers in Little millet (*Panicum sumatranse*) transcriptome. (**c**) Distribution of the number of SSRs repeats, this figure was created using ggplot2 R-package.

MISA software was used to identify potential microsatellites from all 47,358 generated unigenes. A total of 4443 EST-SSRs were discovered (Table S2), and 3593 primers were created (Table S3). The distribution of SSRs in the little millet genome was found to be one SSRs per 10 Kb, comparable to the distribution of SSRs in other cereal genomes^42^. Among the 4,443 EST- SSRs identified in little millet, the tri-nucleotide motif (66.67%) was the most common, followed by di- (27.39%), tetra- (3.83%), Penta- (1.37%), and hexanucleotide motifs (0.71%). This trend demonstrated that the frequency of repeats decreased as motif length increased (See Fig. 3a). The number of microsatellite motif tandem repeats varied from 4 to 98. The most popular microsatellite had eight tandem repeats (1,245, 28.02%), followed by six tandem repeats (890, 20.03%), four tandem repeats (537, 12.08%), and five tandem repeats (465, 10.46%). Microsatellite motifs with more than 24 tandem repeats accounted for just 1.9 percent of the total (Fig. 3b). AG/CT was found to be the amplest type of di-nucleotide repeat, and it was observed to be 59.70% of the repeats, followed by AC/GT (24.50%) and AT/AT (11.10%). Among the tri-nucleotide repeats, the most frequent motifs were AGC/GCT (27.84%) and CCG/CGG (17.65%). The most common tetra-nucleotide repeat motif was AGAT/ATCT (10.52%), whereas the most prevalent Penta nucleotide repeat motif was AGAGG/CCTCT (21.31%). AGGATG/ATCCTC and ACACAG/CTGTGT (15.62%) had the most hexanucleotide motifs (Fig. 3c).

### Microsatellite distribution through genic regions

**Figure 4:**
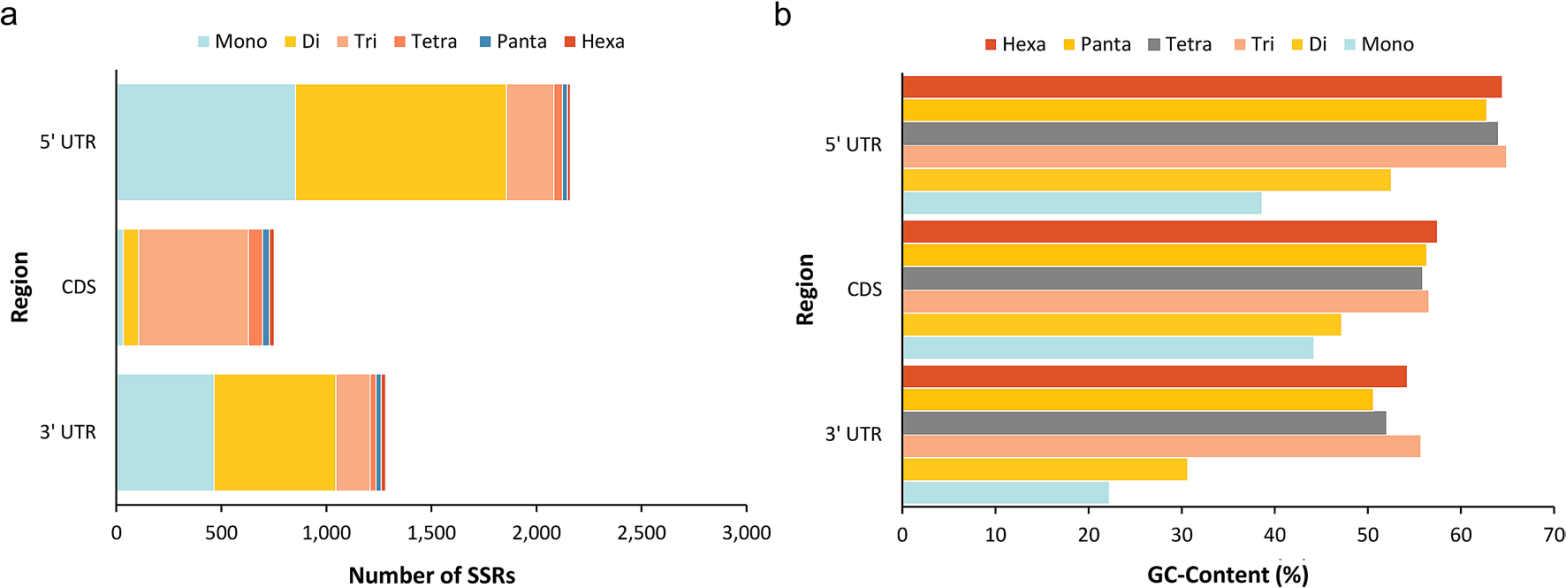
Microsatellite Distribution, (**a**) Distribution of six microsatellite repeat types in different genic regions of Little millet (*Panicum sumatranse*). (**b**) GC-content of mono- to hexanucleotide P-SSRs in different genic regions of Little millet (*Panicum sumatranse*) transcriptome, this figure was created using ggplot2 R-package.

The distribution of microsatellite loci in the transcriptome of little millet was studied. Of the 4,443 genic SSR, 751 and 3442 were located in coding sequence regions (CDS) and untranslated regions (UTRs), respectively (Fig. 4a). The remaining 251 microsatellites were derived from the sample due to a lack of information about their distribution. Microsatellites from different genic regions (CDS, 5′-UTRs, and 3′-UTRs) represented distinct distribution patterns (χ^2^ = 2867.1, P 2.2e−16). The CDS had fewer microsatellites and was dominated by tri-nucleotides (542, 72.17 percent), while the UTRs had a greater density of mono- (1442, 41.89 percent) and di-nucleotide (1585, 46.07 percent) microsatellites. Shorter motif forms (mono-, di-, and tri-nucleotide microsatellites) were found to be more prevalent in the transcriptome (Fig. 4a). Furthermore, there were substantial variations in microsatellite length between three regions (CDS, 5′-UTR, and 3′-UTR), the average length of microsatellites in CDS regions (18.24 bp) was slightly greater than that of UTRs (17.45 bp). (Fig. S2). In these regions, the 5′UTRs had the highest GC content (57.86%) followed by CDSs (52.94%) and 3′UTRs (44.23%). The AT- and GC-content of mono- to hexanucleotide SSRs of these expressed areas were computed, and the findings are shown in Fig. 4b, and Table S4.

### Functional annotation of microsatellites-containing unigenes

To functionally annotate the assembled non-redundant unigenes, a sequence similarity search against the 'nr' database and the Swiss-Prot database was performed using the BLASTx algorithm with cut-off E values of (1e−5) and 1e−10, respectively. According to our findings, 33,160 (69.4%) and 20,686 (43.29%) unigenes were homologous with sequences in the 'nr' and Swiss-Prot databases, respectively. Our result revealed that when subjected to BLAST against 'nr’ database, the majority of the unigenes i.e., more than 78.25 percent of unigenes with lengths greater than 500 bp, matched, whereas 21.75 percent with lengths less than 300 bp matched (Fig. S3).

### GO and KEGG enrichment analysis of microsatellites-containing unigenes

**Figure 5:**
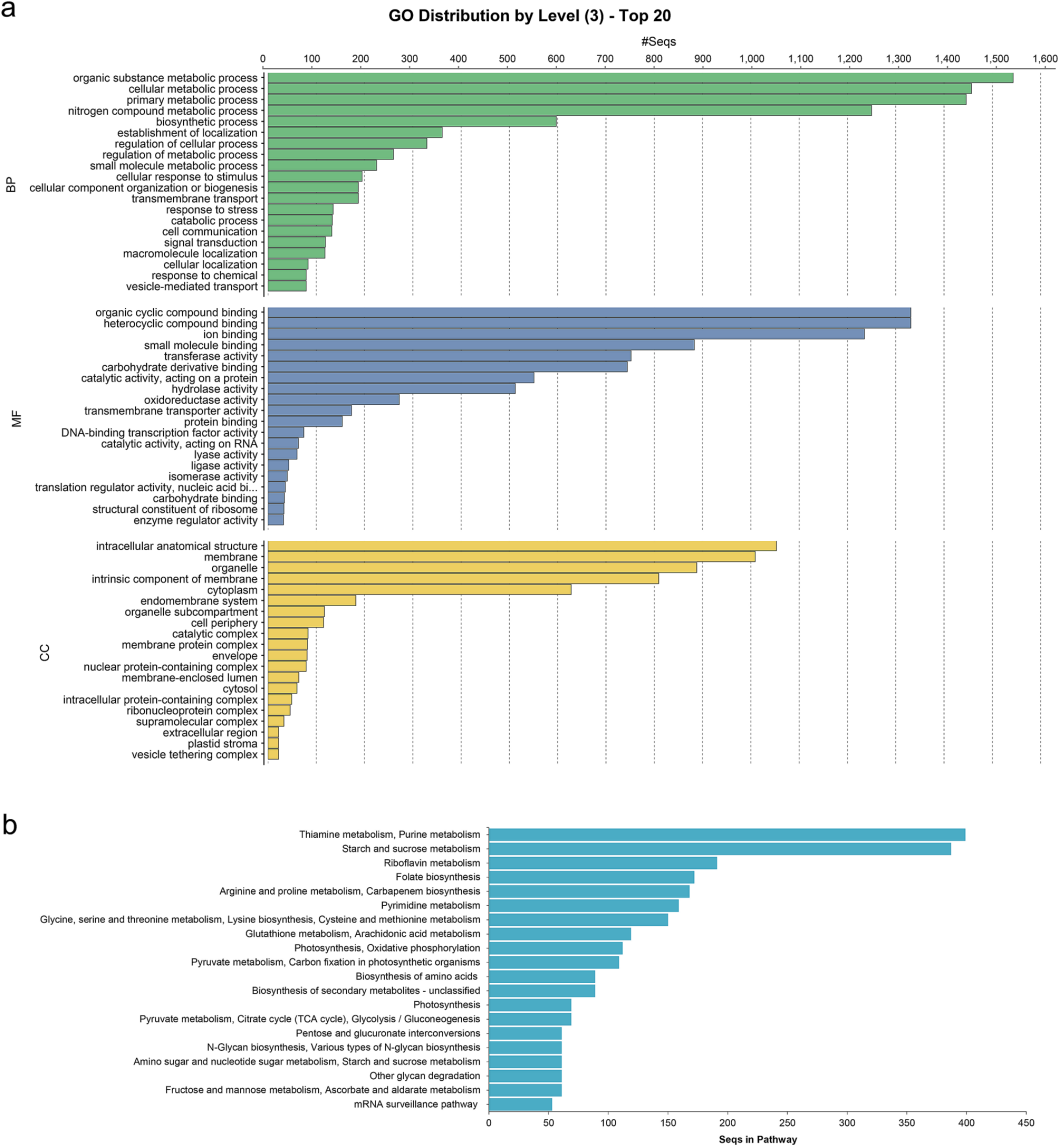
Functions classification of the annotated unigenes. (**a**) Results are grouped by three main functional categories, Biological process, Cellular component and Molecular function. The top abscissa indicates the number of unigenes in a category. Bars show the number of assignments of protein matches to each GO term using BLAST. (**b**) Histogram presentation of KEGG classification, both histograms were created using ggplot2 R-package.

The Blast2GO was used to classify the predicted functions of the assembled unigenes. Figure 5a depicts an overview of the unigene classification in each GO slim expression. GO analysis revealed that 22,717 unigenes (47.96 percent) could be classified into 60 Go terms. Among these unigenes, 8199 (36 P) were classified as Biological Processes, with the most enriched terms being organic substance metabolic process (1,543), cellular metabolic process (1457), and primary metabolic process (1446). The second annotated category was Molecular Function, with organic cyclic compound binding (1331), heterocyclic compound binding (1331), and ion binding (1235) being the most represented GO groups. In the cellular component organization, intracellular anatomical structure (1053), membrane (1009), and organelle (888) were the most represented terms (Fig. 5a and Table S5).

To evaluate the comprehensiveness of our transcriptome library, and thus the efficacy of the annotation protocol, BLASTx was used to align the Unigenes with KEGG, the E-value was kept below 1e−5, and the associated pathways were identified. While 30,400 (60.46%) of the 47,358 unigenes were annotated, only 5,644 (18.56%) were assigned to KEGG pathways. Thiamine and Purine metabolism were the most enriched pathways of the five major KEGG pathways, which included Thiamine metabolism, Purine metabolism (399), Starch and sucrose metabolism (387), Riboflavin metabolism (191), Folate biosynthesis (172), and Pyrimidine metabolism (159). In addition to starch and thiamine metabolism, a plethora of unigenes were involved in the synthesis of other essential vitamins and amino acids, photosynthesis, and secondary metabolite biosynthesis (Fig. 5b and Table S6).

### SSR marker polymorphism assay

The 50 genic SSR primer pairs were randomly chosen from the total number of designed EST-SSR. In total, 39 were amplified and produced expected band size in all five tested minor millets’ species, 24 microsatellite loci showed allelic polymorphism. They were used to analyze the genetic diversity of 25 minor millet genotypes. The observed heterozygosity (Ho) ranged from 0.12 to 0.91, with an average of Ho = 0.49 (Table 2). 217 alleles were obtained from 48 SSR, and the allele rate was 3 to 16 per locus. Most alleles were found at the LtM 38 locus. Other loci with a high number of alleles included LtM 28, LtM 1903, and LtM 103. The average He value obtained was 0.72, which ranged between 0.32 (LtM25) and 0.92 (LtM 3287). The polymorphism percentage obtained for SSR primers ranged from 0 to 100%, with an average value of 91.19 percent per primer. The calculated PIC for each primer ranged from 0.11 to 0.90, with an average of 0.57 for each primer. The SSR primer index (SPI) ranged from 0.5 to 10.2, with an average of 4.21 (Fig. S4 and Table 2). At the species level, barnyard millet showed the largest degree of genetic variation (Na = 5.92, Ne = 3.28, Ho = 0.71, and He = 0.81), while Finger millet had the lowest (Na = 2.67, Ne = 1.44, Ho = 0.44, and He = 0.54) (Table 3).

**Table 2:** Novel genic SSR genetic diversity values in 25 Millet individuals: allele ranges, observed heterozygosity (Ho), expected heterozygosity (He), and (PIC) values of 24 loci.

SSR loci	Repeat motifs	Allele ranges (bp)	Ho	He	PIC	Na	SPI
LtM 10	(CCA)_7_	125–144	0.12	0.36	0.34	3	3.04
LtM 332	(GGAAG)_5_	145–170	0.44	0.86	0.73	5	4.86
LtM 237	(AGC)_8_	158–179	0.55	0.85	0.61	5	5.83
LtM 3287	(GCCTCC)_4_	145–170	0.62	0.92	0.9	5	2.25
LtM 332	(GGAAG)_5_	135–160	0.69	0.68	0.66	5	0.54
LtM 7	(GCG)_6_	146–162	0.43	0.79	0.54	6	9.76
LtM 83	(GGACA)_4_	147–161	0.49	0.76	0.72	6	0.85
LtM 1207	(TGAGCT)_4_	105–153	0.56	0.75	0.72	6	3.05
LtM 340	(TCG)_8_	138–151	0.61	0.8	0.56	6	6.93
LtM 3383	(CCAAT)_4_	140–162	0.47	0.74	0.49	7	5.2
LtM 871	(CTAG)_6_	160–192	0.48	0.76	0.54	7	3.4
LtM25	(CCTC)_5_	120–150	0.31	0.32	0.11	8	3.24
LtM 1808	(CAG)_8_	118–130	0.63	0.87	0.65	8	5.22
LtM 115	(GCT)_5_	110–167	0.41	0.81	0.58	9	4.32
LtM 429	(GATG)_5_	157–179	0.46	0.81	0.77	9	1.02
LtM 32	(CAGAG)_4_	170–240	0.56	0.79	0.53	9	7.08
LtM 259	(GCT)_8_	140–159	0.32	0.63	0.34	10	3.8
LtM 83	(GGACA)_4_	175–195	0.29	0.77	0.52	11	10.2
LtM 1060	(CATGGC)_4_	155–166	0.39	0.58	0.53	11	2.16
LtM 109	(GGC)_7_	100–178	0.4	0.64	0.54	11	2.9
LtM 103	(TTGGA)_4_	148–162	0.27	0.66	0.59	12	0.88
LtM 1903	(ATCTC)_4_	136–150	0.88	0.66	0.41	13	5.04
LtM 60	(AC)_10_	159–171	0.55	0.84	0.6	14	5.94
LtM 28	(GCT)_7_	120–150	0.57	0.88	0.68	15	4.77
LtM 38	(CTG)_7_	130–168	0.91	0.69	0.61	16	3.1
Mean			0.49	0.72	0.57	8.68	4.21

The observed heterozygosity (Ho) ranged from 0.12 to 0.91, with an average of 0.44, whereas the expected heterozygosity (He) ranged from 0.32 to 0.92. (Mean: 0.70).

**Table 3:** Mean of species genetic parameters SSR loci in each of *Millet* species.

Population	No. of alleles	Polymorph allele (%)	Polymorph/monom markers	Na	Ne	Ho	He	PIC
Little Millet	234	97.77	45/1	4.78	2.55	0.49	0.59	0.75
Kodo millet	100	93.61	44/3	3.76	2.24	0.48	0.58	0.5
Barnyard millet	155	87.50	35/5	5.92	3.28	0.71	0.81	0.59
Proso millet	115	77.27	34/10	2.85	1.53	0.58	0.68	0.43
Finger millet	120	87.50	42/6	2.67	1.44	0.44	0.54	0.5

**Figure 6:**
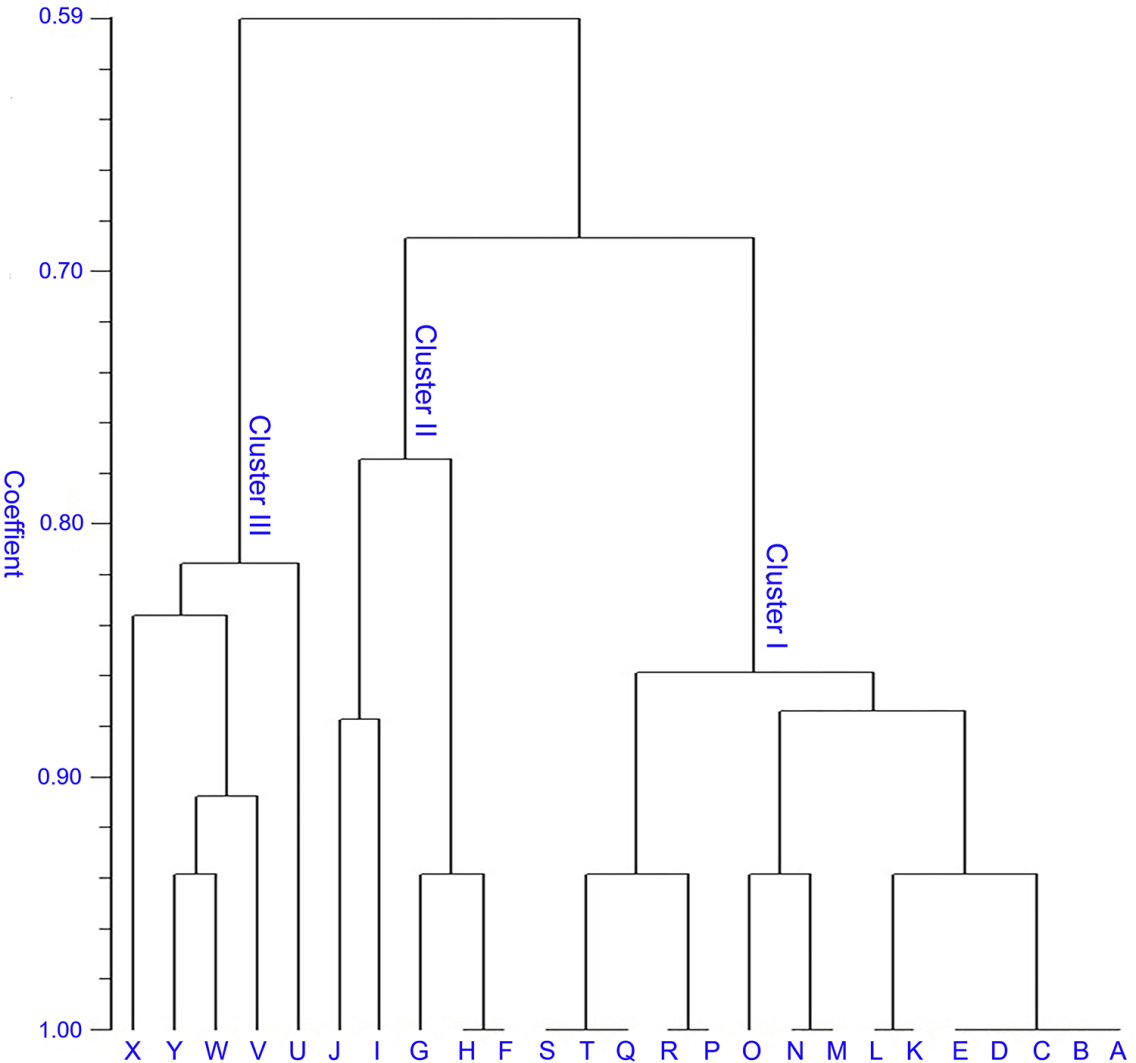
Genetic diversity analysis among miner millet genotypes based on genic SSR markers. The dendrogram shows the genetic relationships among 25 miner millet genotypes, this figure was created using ggplot2 R-package.

The SSR polymorphism data were further used to perform genetic correlation analysis. The genetic similarity matrix was used to generate a dendrogram and accessions. The SSR data of 25 millet genotypes were subjected to similarity index and cluster analysis using Jaccard's coefficient, and UPGMA was performed using the NTSYSpc-2.02i package (Table S7). The dendrogram created using Jaccard's similarity coefficient, and the UPGMA method revealed the highest (80.5 percent) similarity between BAR-1406 and BAR-1407 and the lowest (18.3 percent) similarity between LIT-171 and FIN-1831. The tree plot was created with three main clusters, with an average similarity of 59% (Fig. 6). Cluster I consisted of 15 genotypes of all little millet and Kodo millet genotypes, and it consisted of three sub-clusters, the first sub-cluster of which has two branches, first one includes all little millet genotypes, the second sub-cluster is branched into two, the first one includes two genotypes of barnyard millet, and the second branch includes the remaining genotype, third sub-cluster include Proso Millet genotypes in two branches. The second cluster had two sub-cluster D and E with near about 41% likeness and included Kodo millet genotypes divided between four branches. The last cluster was further subdivided into three sub-cluster consisted of Finger Millet genotypes; among different genotypes, FIN-18301 and FIN-18322 are closely related with 78% similarity, while FIN-1828 has the greatest genetic distance from other genotypes.

### The genes and enzymes involved in the biosynthesis of thiamin

Thiamine (vitamin B1) plays a crucial molecular role for all living organisms. Thiamine diphosphate (ThDP), also known as vitamin B1, serves as an enzymatic cofactor in glucose metabolism, the Krebs cycle, and the biosynthesis branched-chain amino acids in all living organisms. Humans, unlike plants and microorganisms, cannot synthesize ThDP from scratch and must obtain it from their diet. It is shown that severe thiamine deficiency would likely cause the lethal disease beriberi^43^. Therefore, thiamine is a vital micronutrient for humans. Plants are the primary dietary source of thiamine. Little millet is rich in thiamine (0.2–0.48 mg/100 g)^44^, riboflavin (0.12 mg/100 g)^45^, potassium, zinc, magnesium, and manganese minerals, which are essential for healthy human diet^46,47^. According to KEGG analysis results the most enriched pathway was thiamine metabolism; hence, all unigenes were studied and 12 unigenes involved in thiamine metabolism identified (Table 4). Among them, the expression level of *TDPK1*, *TH1*, *THI1, THIC, TPK1, TPK2, PALE1* and *TPK3* were higher in flowering and vegetative than the maturity phase, while transcript level of *TDPC2*, *TENAC*, *THI42* and *TPS1* was higher in maturity in comparison with other samples (Table 4).

**Table 4:** Transcript abundance for candidate genes involved in terpenoids biosynthesis.

Gene	FPKM(V)	FPKM(F)	RPKM(M)	log2(foldchange)V/M	log2(foldchange)F/M
THIC	4898	14,179	3609	0.440593631	1.974084739
THI1	4615	7996	2819	0.71114717	1.504094994
TDPC2	3400	5128	7402	− 1.122380389	− 0.529518874
TPS1	1890	3940	5830	− 1.625109649	− 0.565300254
TPK3	3852	5827	1742	1.144863079	1.742008687
TPK2	4368	5893	588	2.893084796	3.325114206
TENAC	3420	8380	11,106	− 1.699271071	− 0.406317152
THI42	2847	2888	8045	− 1.498649928	− 1.478021679
PALE1	5204	13,843	4184	0.31473811	1.726201875
TH1	3800	11,339	1354	1.48877168	3.065993769
TPK1	3572	9227	1653	1.111645356	2.480774932
TDPK1	4592	8727	2852	0.68714866	1.613511815

## Discussion

### Transcriptome sequencing, assembly, and genic SSR characterization

Minor millets, in particular, little millet, is a highly underappreciated species in terms of popularity and research interest, despite being a traditional food and fodder crop with a high nutrient profile. Millet molecular breeding is hampered by a lack of literature on molecular breeding and the inaccessibility of co-dominant markers. The identification and use of eSSR markers to screen accessions, varieties, germplasm, or cultivars will speed up the breeding process^48^. Transcriptome sequencing is a sophisticated and efficient method to detect new genes, identifying expression patterns, and developing molecular markers^49^. In this research, the Ion torrent S5 technology platform was utilized, and a total of 30 million reads (about 15 GB) were generated from three different samples. For sequence annotation, BLASTx against the NCBI database's ‘nr' protein revealed that 57.30% (27,340) of the 47,358 unigenes in our dataset had at least one significant homologous gene in other species, and approximately 42.7 percent of unigenes could not be functionally annotated either because they performed similar properties as protein with an uncharacterized function or there were no BLAST matches. In most cases, the length of the query sequence influences the ability to discover essential similarities between the sequences. Several previous studies found that the likelihood of BLAST matches in protein databases could be raised while increasing the unigenes length^50^. This was also perceived in the current research, where 80 percent of the unigenes with lengths greater than 500 bp had homologs, whereas only 20 percent of the unigenes with lengths less than 300 bp were detected be similar to other homologs. Furthermore, there is limited data on the transcriptome, genome, and genes of little millet; in consequence, numerous little millet genes are not publicly available.

The scarcity of SSR markers limits basic and applied genomics research in little millet. Transcriptome sequencing generates a massive amount of sequence data, which can be used to create a large number of SSRs. Markers derived from transcriptomic sequences are far more helpful than markers derived from genomic sequence data for gene-based interpretation and detecting functional variation^51^. A total of, 9764 potential EST-SSR markers were identified from 47,358 unigene sequences. The genic microsatellites frequency was 11.25 percent, and the distribution density was one SSR per 10 kb, which was remarkably higher than sesame (8.9%), barley (2.8%), Asian lotus (8%), maize (7%), but lower than cotton (12%)^23,52–55^. Some variability in the data mining method, search parameters, and scale of the unigene assembly dataset maybe addressed by variations in SSR abundance^56^. Here we discovered six distinct repeat motifs. Tri-nucleotide repeats (66.67percent) were the most prevalent type, followed by di-nucleotide repeats (27.39 percent) and tetra- nucleotide (3.83 percent). The AGC/GCT motif (825, 30.99 percent) was the most common of the tri-nucleotide repeats, followed by the CCG/CGG motif (523, 17.52 percent). AG/CT (726, 20.44 percent) was the most dominant motif among the di-nucleotide repeats, which was similar with study in *Triticum aestivum* L.^57^, and *Raphanus sativus* L.^58^. In comparison, the CG/CG motif had the lowest frequency.

### Genetic relationship, polymorphism, and transferability of EST-SSR markers

The majority of reports on genetic diversity analyses in little millet germplasm have used RAPD^59^, SNP and genomic SSR markers^60^. Tiwari, et al.^59^ using 60 RAPD investigated the diversity of 36 little millet (*Panicum sumatrense*) genotypes, although these researchers observed high polymorphisms among the markers used, but as RAPD technique is notoriously laboratory dependent their results are hardly reproducible in other laboratory or in other genotypes. And there are only a few reports of EST-SSR application in little millet. Ali et al.^61^ using 22,961 EST sequences of switchgrass (*Pancium virgatum*) developed 48 species transferable EST-SSR markers. Das et al.^62^ also applied salinity response transcriptome of *P. sumatrense* to develop 37,100 genic SSR markers, that might be associated to salinity and drought stress tolerance. Genic SSRs are advantageous and frequently preferred for coding regions of the genome. Furthermore, the degree of transferability of this type in related species can provide a high level of acceptability^63^. In this research using transcriptome sequencing of samples of three developmental stages, species transferable genic SSR marker sets were mined. To verify generated SSR markers, 50 primer pairs were randomly selected and validated, 39 of which were positively amplified in 25 millet genotypes (Table S1). The lack of amplicons from other primer pairs is most likely due to primer positioning across large introns, splice sites, or poor-quality sequences^64^. Because of more significant sequence conservation in the transcribed regions, the rate of polymorphism of genic SSRs is typically lower than that of genomic SSRs^65^. However; this was not the case in this research, because the genic SSRs discovered were highly polymorphic. The average number of alleles recorded in this research using genic SSR markers was 8.68 per locus, with values ranging from 3 to 16 alleles. Among the 39 primer pairs tested in our study, 24 (61 percent) exhibited polymorphism. The determined value was greater than the polymorphism recorded by Senthilvel et al.^66^ in the examined pearl millet varieties. Polymorphism variation is related to the geographic origins of specimens, the number of samples, the genome sequence of the samples, and the primers used^67^. In addition, the average PIC value was higher (0.57) as the result of the greater depth of sequencing coverage achieved in this research. One of the critical factors influencing polymorphism is the length of the microsatellite. SSR length may be classified as short (12 bp), medium (12–20 bp), or high (> 20 bp)^68^. In this study, medium SSR lengths (2,055, 46.24 percent) had the greatest proportion. Thus, the microsatellites created from little millet transcriptome probably show a moderate degree of polymorphism. Six microsatellite motifs show high variations in length (Kruskal–Wallis rank-sum test, P 1.8 e−16), and the frequency of each decreased with increasing motif size (Nemenyi test, P 2.2e−16). Using five populations of minor millets, we confirmed the polymorphism of the 48 microsatellite markers. A total of 217 alleles were found. At the species level, Barnyard millet and Finger millet showed the highest and the lowest genetic diversity, respectively. Cluster analysis divided genotypes into three main clusters, with an average similarity of 59 percent. Cluster-I contains two species, little millet, and Kodo millet, with 62 percent likeness. Cluster-II has Kodo millet genotypes and consists of four branches with a 58 percent likeness. The third cluster, which is divided into three sub-clusters, contains Finger Millet genotypes. Genetic variation in species is affected by internal and external (historical or evolution causes) factors, little millet-as an ancient plant, has collected a large number of genetic variants during the long-term evolution, and it is supposed to retain a high degree of genetic diversity. In this study at the population level, all five minor millet species showed low genetic diversity, which could be a result of a small number of members in each population and distribution properties. Kodo millet showed the largest genetic diversity among all studied spices. While Barnyard millet and Proso Millet revealed limited genetic diversity, which could be the reason of gene flow between the two species during evolution. Generally, Microsatellite markers derived from transcriptome data/ESTs have a higher degree of transferability in related species^69^. In the current study, 39 microsatellite markers were successfully amplified in minor millets, with 24 of them being polymorphic in all minor millets’ genotypes. The transferability ratio was 78%, which was higher than the transferability ratios recorded in Pistacia^26^ but less than Moon Seed^70^ and Arrowhead^71^. Genic SSRs generated in this research have strong potential for cross-species amplification and could be utilized in future genetic studies of other cereals species.

### Potential function of unigenes containing microsatellites

Microsatellites derived from transcribed sequences can be closely associated with gene function and thereby may have important influences on gene products, causing phenotypic changes, and controlling gene expression. To expose the possible roles of these unigenes, functional annotations and classification of microsatellites-containing unigene sequences are performed. According to GO functional annotations, a significant amount of unigenes, including microsatellites were assigned to terms such as "regulation of cellular process, cellular metabolic process, biosynthetic process, cell communication catalytic action" and response to stress, implying that they could be associated with little millet’s basic metabolism, growth and developmental activities. Likewise, KEGG analyses revealed that SSRs, including unigenes, served various biological roles and were involved in different vital features of little millets. Notably, thiamine metabolism and purine metabolism were the most enriched pathway in KEGG analysis. Looking deeper, we identified twelve genes involved in the thiamine biosynthetic pathway. Compared to the maturity stage, expression levels of genes involved in the vitamin B1 pathway were higher during the vegetative and flowering phases. These results are in agreement with the report of Colinas and Fitzpatrick, 2015, where the expression of genes involved in thiamine biosynthesis was higher in photosynthesizing tissues^72^. Most of the genes involved in thiamine biosynthesis are not fully known; hence to improve human health and nutrition, it is vital to identify the genes and enzymes involved in thiamine biosynthesis^73^.

## Conclusions

Using transcriptome data, a total of 4443 new SSR markers were developed, and the frequency, distribution, and function of these genic microsatellites were characterized. The unigenes containing microsatellites performed a spread spectrum of biological roles, the majority of which were related to thiamine metabolism, purine metabolism, other metabolic process, and signaling pathways. These findings shed light on the function of microsatellites in the transcriptome. Furthermore, the polymorphism and transferability of 24 eSSR markers were examined in 25 minor millet genotypes. The results of this research can present an excellent resource for germplasm identification, genetic relationship studies, linkage maps, MAS reproduction, and diversity analysis in little millet and other related species in future genetic and genomic studies, as well as play a substantial role to update the millet genic SSR markers database.

## Supplementary Information

Supplementary Legends.

Supplementary Figure S1.

Supplementary Figure S2.

Supplementary Figure S3.

Supplementary Figure S4.

Supplementary Tables.

## Data Availability

Transcriptome data generated in this study were submitted to NCBI with the SRA ID SRR14509267, SRX10855102, SRR14509268, SRX1085510, SRR14509269, SRX10855100, and SAMN19114419.
