# Supplementary material for: Genic microsatellite marker characterization and development in little millet (Panicum sumatrense) using transcriptome sequencing
Source: Sci Rep. 2021 Oct 18;11:20620. doi: 10.1038/s41598-021-00100-4 (PMC8523711; doi:10.1038/s41598-021-00100-4)
Supplement: Supplementary file 1 — Supplementary Legends. [file 41598_2021_100_MOESM1_ESM.docx]

**Fig S1. Size distribution of reads.** Length distribution of the sequencing reads after trimming low-quality reads. This fig was created using ggplot2 R-package.

**Fig S2.** Box plots of the length distribution of microsatellite loci located in different genic regions. This fig was created using ggplot2 R-package.

**Fig S3.** Number of Go term for sequence with different length. This fig was created using ggplot2 R-package.

**Fig S4.** SSR validation for 25 millet genotypes

**Table S1: List of genotypes for SSR primer validation**

**Table S2. Detailed parameters of the SSRs identified in the transcriptome**

**Table S3: Primer sequences of 3795 EST-SSR markers of little millet (*Panicum* *sumatranse*)**

**Table S4. The AT-content (%) of mono- to hexanucleotide P-SSRs in the different genic regions**

**Table S5: GO classification of unigens containing eSSR**

**Table S6: Pathways identified by functional classification using KEGG**

**Table S7: Jaccard’s similarity coefficient of 25 minor millet genotypes based on eSSR**
