# Supplementary figures and images for: Genic microsatellite marker characterization and development in little millet (Panicum sumatrense) using transcriptome sequencing
Source: Sci Rep. 2021 Oct 18;11:20620. doi: 10.1038/s41598-021-00100-4 (PMC8523711; doi:10.1038/s41598-021-00100-4)

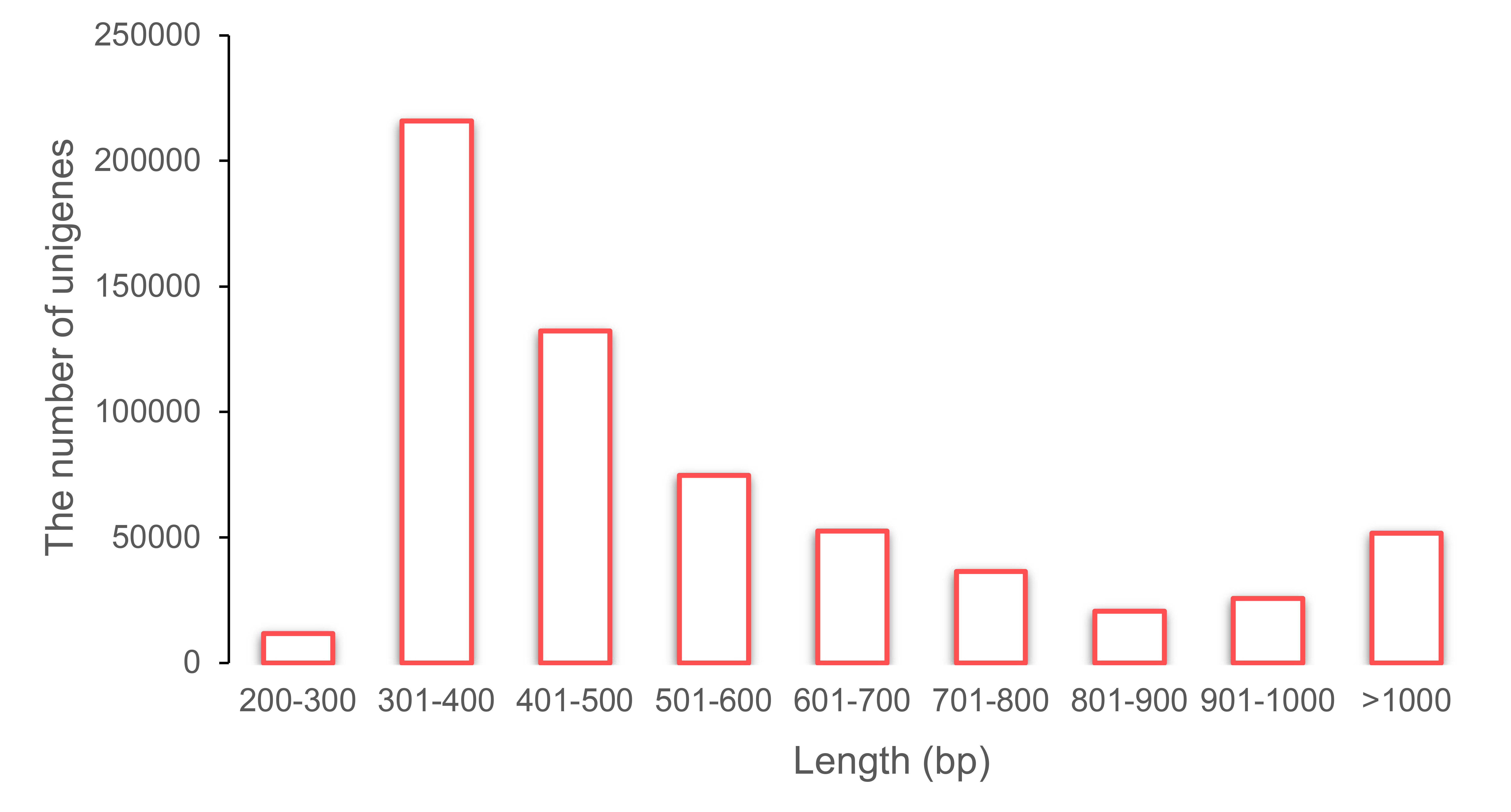

Supplement: Supplementary file 2 — Supplementary Figure S1. [file 41598_2021_100_MOESM2_ESM.jpg]

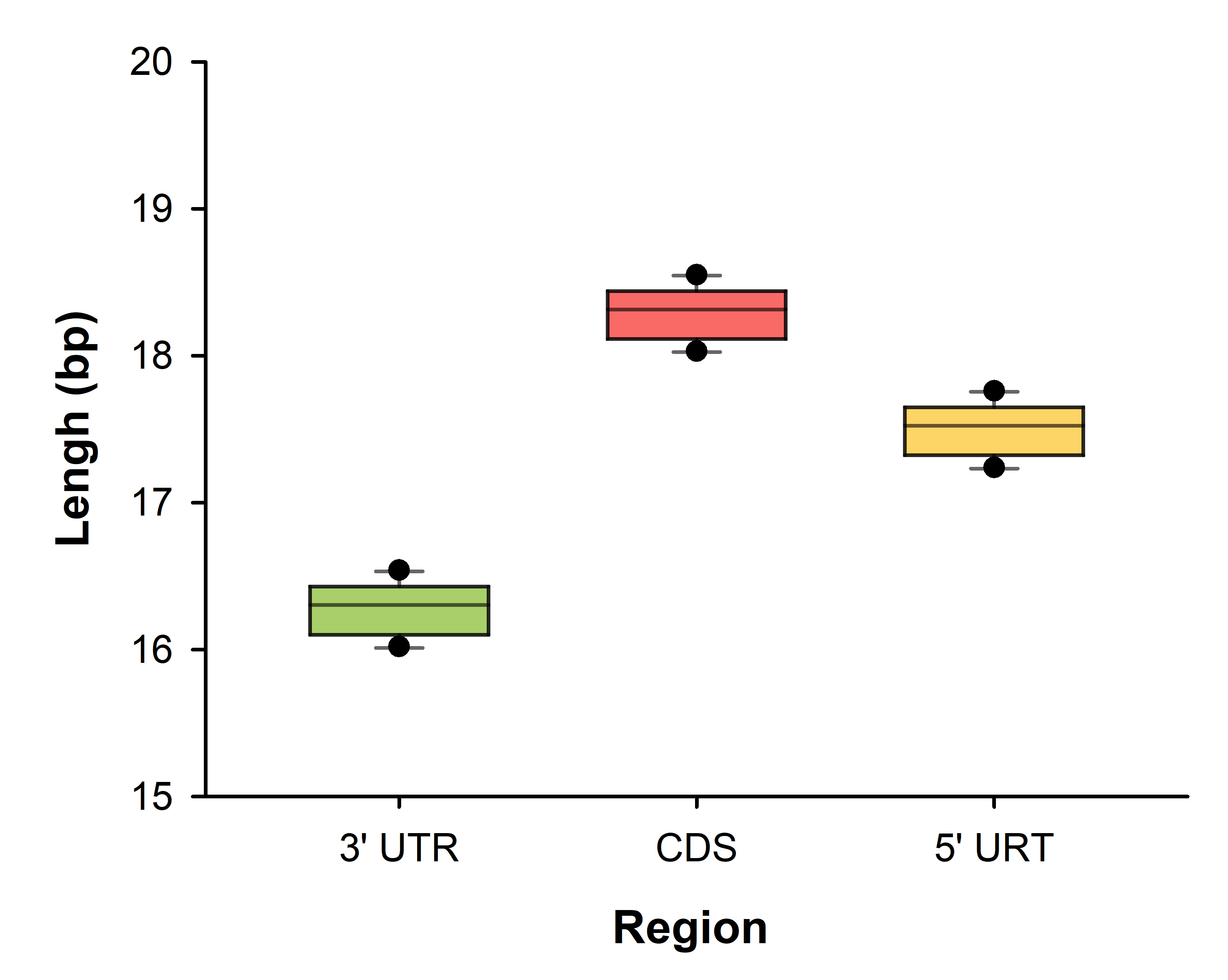

Supplement: Supplementary file 3 — Supplementary Figure S2. [file 41598_2021_100_MOESM3_ESM.jpg]

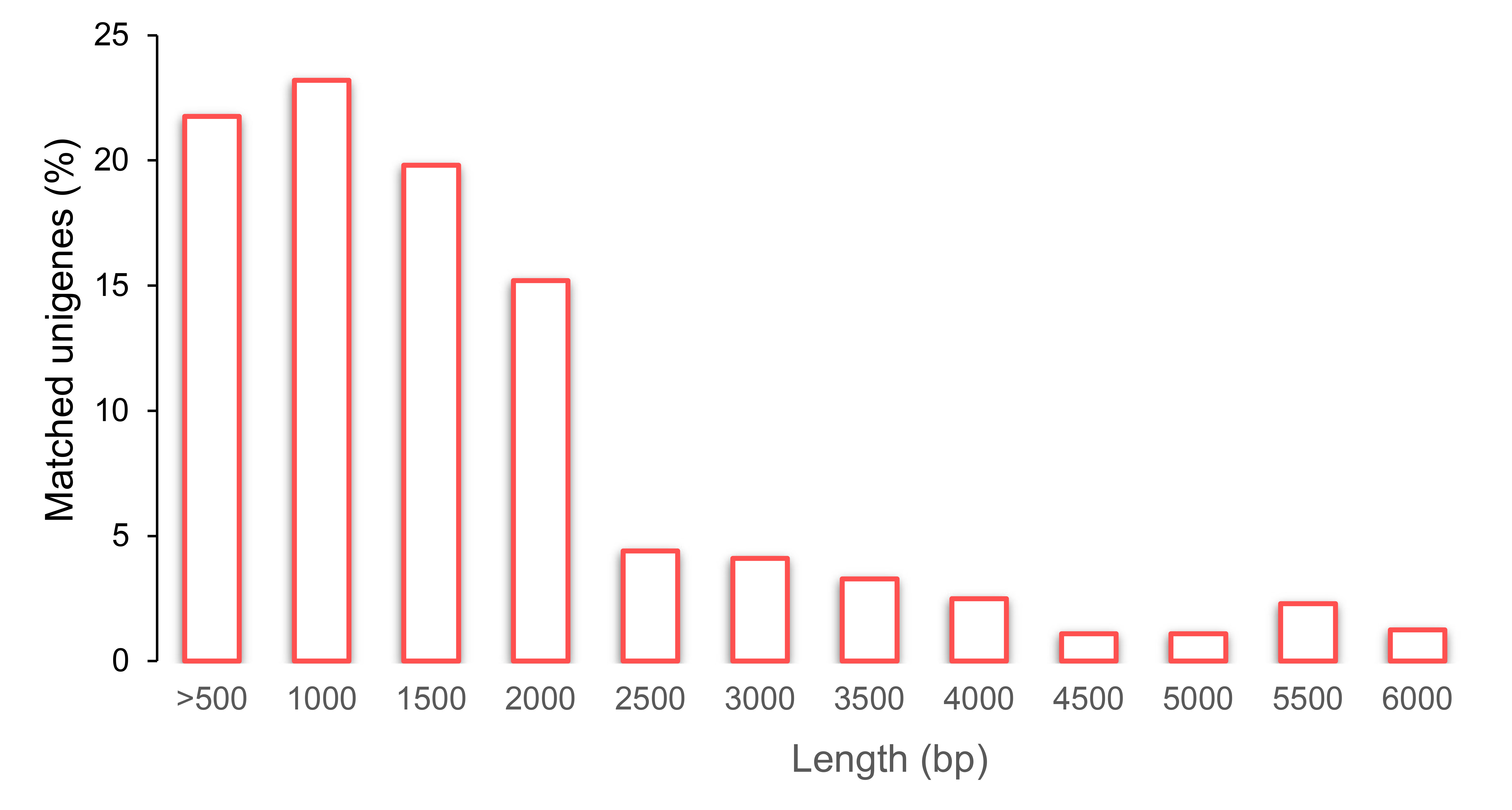

Supplement: Supplementary file 4 — Supplementary Figure S3. [file 41598_2021_100_MOESM4_ESM.jpg]
